# Desmopressin and bleeding risk after percutaneous kidney biopsy

**DOI:** 10.1186/s12882-019-1595-4

**Published:** 2019-11-15

**Authors:** Ambarish Athavale, Hemant Kulkarni, Cagil D. Arslan, Peter Hart

**Affiliations:** 1 Division of Nephrology, Cook County Health, 1950 W. Polk Street, 5th Floor, Chicago, IL 60605 USA; 2 M&H Research, LLC, San Antonio, TX USA

**Keywords:** Desmopressin, Bleeding, Kidney biopsy, CKD

## Abstract

**Background:**

Desmopressin is used to reduce bleeding after kidney biopsy but evidence supporting its use is weak, especially in patients with elevated creatinine. The present study was undertaken to evaluate efficacy of desmopressin in reducing bleeding after percutaneous kidney biopsy.

**Methods:**

Retrospective cohort study. 269 of 322 patients undergoing percutaneous kidney biopsy between January 1, 2014 and January 31, 2018 were included. Patients had normal bleeding time, platelet count and coagulation profile. Primary outcome was defined as composite of hemoglobin drop ≥1 g/dL, hematoma on post biopsy ultrasound, gross hematuria, erythrocyte transfusion or angiography to stop bleeding. Association of desmopressin with outcomes was assessed using linear (for continuous variables) and logistic (for binary variables) regression models. Propensity score was used to minimize potential confounding.

**Results:**

Desmopressin was administered to 100/269 (37.17%) patients. After propensity score adjustment patients who received desmopressin had increased odds of post biopsy bleeding [OR 3.88 (1.95–7.74), *p* < 0.001]. Creatinine at time of biopsy influenced bleeding risk; gender, emergent vs elective biopsy, obesity, AKI, diabetes, hypertension or bleeding time did not influence bleeding risk. Administration of desmopressin to patients with serum creatinine ≥1.8 mg/dL decreased bleeding risk [OR 2.11 (95% CI 0.87–5.11), *p* = 0.09] but increased bleeding risk when serum creatinine was < 1.8 mg/dL (OR 9.72 (95% CI 2.95–31.96), *p* < 0.001).

**Conclusion:**

Desmopressin should not be used routinely prior to percutaneous kidney biopsy in patients at low risk for bleeding but should be reserved for patients who are at high risk for bleeding.

## Background

Percutaneous kidney biopsy is the gold standard for diagnosis of kidney disease but is associated with bleeding complications such as macroscopic hematuria (3.5%), post biopsy hematoma (11.6%), erythrocyte transfusion (0.9%) and rarely nephrectomy (0.01%) or death (0.02%) [1]. Incidence of bleeding after kidney biopsy range from 13 to 34% [2, 3]. The incidence of bleeding is increased when biopsy is performed in patients with bleeding diathesis, advanced age, female sex, anemia, uncontrolled hypertension, elevated serum creatinine or acute kidney injury (AKI) and in hospitalized patients [1, 3–5]. However, no patient characteristic or laboratory test can reliably predict bleeding post kidney biopsy. Desmopressin (DDAVP) has been shown to improve platelet function and decrease bleeding due to uremia [6, 7]. As a result, desmopressin has been routinely used to prevent bleeding after kidney biopsy. Manno et al. reported decreased risk of bleeding and hematoma size in a randomized controlled trial of patients undergoing percutaneous kidney biopsy [8]. However, patients with serum creatinine > 1.5 mg/dL or eGFR < 60 ml/minute and patients with acute kidney injury (AKI) were excluded from this study. Decreased bleeding risk reported by Manno et al. was primarily from decrease in asymptomatic post biopsy hematoma and desmopressin did not decrease any clinically relevant endpoints (such as erythrocyte transfusion, angiographic intervention to stop bleeding etc). Thus, it is not clear if desmopressin should be administered routinely prior to kidney biopsy in patients with preserved kidney function and effectiveness of desmopressin in preventing bleeding complications in patients with decreased kidney function has not been well studied.

The aim of this study was to evaluate the effect of desmopressin in reducing bleeding complications after percutaneous kidney biopsy particularly in patients with decreased kidney function.

## Method

### Study overview

This is a retrospective study of consecutive patients undergoing percutaneous kidney biopsy at Cook County hospital between January 2014 and January 2018. The institutional review board at Cook County Health approved the study protocol.

### Participants, setting and intervention

All consecutive patients undergoing kidney biopsy during the study period were included in the study. Kidney biopsy was performed under real time ultrasonographic guidance with an 18-gauge end-cut spring mounted needle. Patients with bleeding time > 10 min, platelet count < 50,000 or coagulopathy (INR > 1.5 or PTT > 40 s) were excluded from the study. Aspirin was discontinued for 1 week, clopidogrel for 5 days and anticoagulation [(warfarin, heparin, novel oral anticoagulant (NOAC)] was stopped as indicated. Clinical and demographic information was obtained from the patients’ medical record. A biopsy was deemed to be emergent if performed to evaluate an AKI or rapidly progressive glomerulonephritis. All kidney biopsies were performed by attending Nephrologists. All patients had blood pressure controlled to < 140/90 prior to the biopsy. The decision to administer desmopressin (0.3 mcg/kg intravenous, 30 min prior to biopsy) was per the Nephrologist performing the biopsy. A baseline hemoglobin was obtained for every patient within 24 h prior to kidney biopsy and hemoglobin was checked 6 h and between 12 and 24 h after kidney biopsy as per hospital protocol. An ultrasound was performed immediately after the kidney biopsy for presence of hematoma after the biopsy. A follow-up ultrasound was performed after 24 h if patient had drop in hemoglobin > 1 g/dL, significant post biopsy pain or gross hematuria. Adverse events or side effects of desmopressin were noted in patient medical record. All patients were monitored for 24 h after the kidney biopsy as per hospital protocol.

### Objectives

The objective of the study was to evaluate if desmopressin decreases risk of post biopsy bleeding in patients undergoing percutaneous kidney biopsy. We also evaluated whether efficacy of desmopressin was similar in patients with decreased kidney function and patients with normal kidney function.

### Outcomes

Primary outcome was any bleeding event which was defined as composite of hemoglobin drop ≥1 g/dL, hematoma on post biopsy ultrasound, gross hematuria, need for angiogram or need for red blood cell transfusion. Secondary outcomes were post biopsy decrease in hemoglobin ≥1 g/dL, hematoma on post-biopsy ultrasound, need for erythrocyte transfusion, angiographic procedure to stop bleeding or hypotension.

### Statistical analysis

Descriptive statistics included mean (SD) for continuous variables and number (%) for categorical variables. Differential distribution of the baseline characteristics across the study arms was tested for statistical significance using Student’s T test for continuous variables and Pearson’s chi-square test for categorical variables. When the cell values were below 5, we used Fisher’s exact test in place of the chi-square test. Since this dataset represents the contemporary practice of prebiopsy DDAVP use, we anticipated some confounding by indication to be operational. To account for the known and putative confounders, we used propensity score adjustment analyses. Baseline and intraprocedural characteristics that were different across study arms at a statistical significance < 0.1 were included as potential predictors of DDAVP use in a logistic regression framework. Based on this model, a propensity score was derived using the software prop_sel for Stata package. The appropriateness of the propensity score was examined using standardized differences across study groups before and after adjustment with a goal to converge these differences between − 0.1 and 0.1. Also, receiver-operating characteristic curve (ROC) was generated and area under this curve (AUROC) was used as an indicator of the accuracy of the propensity score to predict the likelihood of DDAVP use. This propensity score was used as a covariate in all association analyses that determined the strength of association of DDAVP use with study outcomes.

Association analyses were conducted using a linear (for continuous variables) or logistic (for dichotomous variables) regression that accounted for the baseline differences via the propensity score. Effect sizes used were mean difference for continuous variables and odds ratios for dichotomous variables. Association of DDAVP use with bleeding was investigated on the backdrop of several potential effect modifiers. For these analyses, we estimated the odds ratio for association of DDAVP use with bleeding within each category of the effect modifier (e.g. in males and females separately). Since such analyses lead to multiple hypothesis testing, we corrected the statistical significance by the false discovery rate (FDR) correction using the Benjamini-Hochberg method. Statistical significance was assessed with a global type II error rate of 0.05. All statistical analyses were conducted using the Stata 12.0 package (Stata Corp, College Station, TX).

## Results

**Fig. 1:**
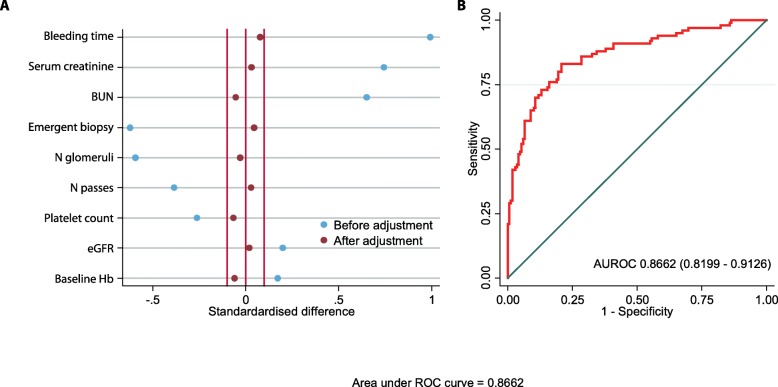
Propensity score matching and area under the ROC curve for propensity score. Blue dots represent distrubution of variables before matching and red dots after matching. **a** Standardized difference before and after adjustment for propensity score. **b** Area under ROC curve for propensity score to predict the likelihood of DDAVP use

From January 2014 to January 2018, 322 patients underwent percutaneous kidney biopsy. Fifty-three patients were excluded because bleeding time was not available on these patients and 269 patients for whom all data was available were included in the study. Baseline demographic and clinical variables are included in Table 1. One hundred (37.17%) patients received desmopressin. Patients who received desmopressin were similar to patients who did not receive desmopressin by age, sex, race/ethnicity, emergent biopsy, diabetes, hypertension, body mass index, blood pressure, PT/PTT, number of passes and indication for biopsy. The desmopressin group had lower hemoglobin at baseline (10.38 ± 1.76 vs 11.51 ± 1.89, *p* < 0.001), lower platelet count (214.82 ± 67.82 vs 245.02 ± 87.88, *p* = < 0.001), lower eGFR (38.56 ± 38.81 vs 64.93 ± 49.59, *p* < 0.001) and higher bleeding time (7.21 ± 2.81 vs 4.77 ± 2.06, *p* < 0.001), higher blood urea nitrogen (43.60 ± 25.67 vs 29.22 ± 17.78, *p* < 0.001) and serum creatinine (3.34 ± 2.60 vs 1.79 ± 1.38, *p* < 0.001) as compared to no desmopressin group. As described above, propensity score was generated to account for variables that were significantly distributed in the two groups (Fig. 1). The AUROC for the propensity score was 0.86 (0.81–0.91).

**Table 1:** Descriptive characteristics of the patients enrolled in this study (*N* = 269). Numbers indicate N and % for categorical variables and Mean and SD for continuous variables. *P* values are reported from Student’s t test for continuous variables and Pearson’s chi-square test for categorical variables

Characteristic	Desmopressin given		Desmopressin not given		P
	Mean / N	SD / %	Mean / N	SD / %	
Age (y)	46.05	14.44	48.22	13.62	0.22
Females	55	55.00	89	52.66	0.71
Hispanic ethnicity	62	62.00	86	50.89	0.08
Race					0.28
African American	52	52.00	65	38.46	
White	32	32.00	71	42.01	
Asian	2	2.00	5	2.96	
American Indian/ Alaska Native	1	1.00	1	0.59	
Other	13	13.00	27	15.98	
Emergent biopsy	26	26.00	30	17.75	0.11
Diabetes	38	38.00	56	33.14	0.42
Hypertension	81	81.00	140	82.84	0.70
Body mass index (Kg/m^2^)	29.35	7.01	30.05	6.80	0.42
Systolic blood pressure (mmHg)	133.15	15.99	134.06	18.21	0.68
Diastolic blood pressure (mmHg)	76.00	10.19	76.45	10.85	0.74
Hemoglobin (g/dl)	10.38	1.76	11.51	1.89	< 0.001
Platelet count (× 10^3^/μl)	214.82	67.82	245.02	87.88	0.00
Platelet functional assay - Epinephrine (s)	141.83	65.72	152.76	54.45	0.57
Platelet functional assay - ADP (s)	102.83	32.12	106.26	41.10	0.79
Prothrombin time (min)	13.62	1.10	13.58	1.40	0.80
INR	1.05	0.11	1.04	0.11	0.26
Partial thromboplastin time (min)	30.68	4.12	31.42	6.10	0.28
Bleeding time (min)	7.21	2.81	4.77	2.06	< 0.001
Blood urea nitrogen (mg/dl)	43.60	25.67	29.22	17.78	< 0.001
Serum creatinine (mg/dl)	3.34	2.60	1.79	1.38	< 0.001
eGFR (ml/min)	38.56	38.81	64.93	49.59	< 0.001
Need for RBC treatment	5	5.00	1	0.59	0.02
Need for FFP	2	2.00	1	0.59	0.29
Number of glomeruli	20.96	9.27	19.36	9.16	0.17
Number of passes	2.58	0.10	2.88	0.10	0.05
Indication for biopsy					
Nephrotic syndrome	42	42.00	76	44.97	0.64
Nephritic	53	53.00	69	40.83	0.05
Chronic hematuria	2	2.00	6	3.55	0.47
Acute kidney failure	5	5.00	5	2.96	0.39
Transplant kidney	7	7.00	17	10.06	0.39
Chronic kidney disease	35	35.00	50	29.59	0.36

Primary outcome was seen in 59.46% patients who received desmopressin as compared to 31.75% who did not receive desmopressin (*p* < 0.001). After adjustment for propensity score patients who received desmopressin continued to have increased odds of post biopsy bleeding [OR - 3.88 (1.95–7.74), *p* < 0.001]. The primary outcome was driven mainly by post biopsy decrease in hemoglobin (44.14% vs 18.96%) and post biopsy hematoma (18.02% vs 15.17%) in patients who received desmopressin. After propensity score matching, episodes of gross hematuria and need for red blood cell (RBC) transfusion were similar in the two groups (Table 2). We performed sub-group analysis to determine factors contributing to increased bleeding seen in patients who received desmopressin. On sub-group analysis, high baseline eGFR and low serum creatinine were associated with increased bleeding risk. Gender, emergent vs elective biopsy, obesity, acute kidney injury, diabetes, hypertension or bleeding time did not influence bleeding risk (Table 3). Administration of desmopressin to patients with high creatinine (≥ 1.8 mg/dL) decreased bleeding risk (OR 2.11 (95% CI 0.87–5.11), *p* = 0.09) while administration of desmopressin to patients with low baseline creatinine (< 1.8 mg/dL) was associated with a very high bleeding risk (OR 9.72 (95% CI 2.95–31.96), *p* < 0.001). There were no adverse events or side effects attributed to desmopressin and no patient had symptomatic hyponatremia.

**Table 2:** Association of the study outcomes with DDAVP use

Outcome	DDAVP Arm		No DDAVP Arm		P	ES	95% CI	P_adj_
	Mean / N	SD / %	Mean / N	SD / %				
Post Biopsy SBP (mmHg)	129.89	16.13	129.91	18.27	0.99	−1.02	−6.83 - 4.79	0.73
Post Biopsy DBP (mmHg)	73.64	10.56	75.13	10.74	0.23	−3.55	−6.96 - -0.14	0.04
ΔSBP (mmHg)	2.77	12.21	4.15	13.79	0.38	−2.93	−7.24 - 1.38	0.18
ΔDBP (mmHg)	2.78	10.31	1.85	8.86	0.40	−0.27	−3.16 - 2.62	0.86
Post Biopsy Hemoglobin (g/dl)	9.48	1.71	11.17	1.82	< 0.001	−0.64	−1.18 - -0.11	0.02
ΔHemoglobin (g/dl)	0.90	0.82	0.36	0.76	< 0.001	0.58	0.32–0.83	< 0.001
Hb drop by ≥1 g/dl	49	44.14	40	18.96	< 0.001	3.44	1.69–6.99	< 0.001
Hematoma	20	18.02	32	15.17	0.51	2.53	1.08–5.94	0.03
Hematuria	10	10.64	6	3.17	0.01	5.12	0.77–34.09	0.09
RBC transfusion needed	5	4.50	1	0.47	0.02^a^	1.18	0.09–16.32	0.90
Bleeding event	66	59.46	67	31.75	< 0.001	3.88	1.95–7.74	< 0.001
Length of stay (d)	5.23	6.82	3.02	3.02	< 0.001	0.89	−0.49 - 2.27	0.20

*SBP* Systolic blood pressure, *DBP* Diastolic blood pressure, *ES* Effect size – represented as mean difference for continuous variables and odds ratio for dichotomous variables; P_adj_ – Significance value after adjusting for the propensity score; ^a^, due to small cell numbers Fisher’s exact test was used

**Table 3:** Subgroup analyses to assess modifier influence on the association of DDAVP with bleeding

Effect Modifier	Category	N	OR	95% CI	P	q
Gender	Male	125	2.82	1.01–7.83	0.05	0.28
	Female	144	5.14	1.97–13.47	< 0.001	0.02
Emergency biopsy	No	213	5.28	2.27–12.31	< 0.001	0.003
	Yes	56	1.87	0.53–6.60	0.33	0.52
Obese	No	153	3.40	1.34–8.65	0.01	0.12
	Yes	116	4.74	1.67–13.44	0.003	0.06
CVD	No	253	3.76	1.86–7.58	< 0.001	0.006
	Yes	13	1.00	. - .	.	.
AKF	No	259	3.87	1.91–7.83	< 0.001	0.005
	Yes	10	3.33	0.08–131.75	0.52	0.52
CKD	No	184	4.29	1.91–9.64	< 0.001	0.01
	Yes	85	2.81	0.73–10.79	0.13	0.40
Diabetes	No	175	3.44	1.49–7.91	0.004	0.06
	Yes	94	5.47	1.54–19.42	0.009	0.11
Hypertension	No	48	4.33	1.10–17.10	0.04	0.28
	Yes	221	3.59	1.59–8.09	0.002	0.04
Bleeding time^a^	Low	137	3.68	1.34–10.07	0.01	0.12
	High	132	4.22	1.61–11.07	0.004	0.06
Baseline eGFR^a^	Low	136	2.32	0.97–5.55	0.06	0.29
	High	133	8.45	2.60–27.46	< 0.001	0.01
Baseline creatinine^a^	Low	140	9.72	2.95–31.96	< 0.001	0.005
	High	129	2.11	0.87–5.11	0.10	0.40
Prothrombin time^a^	Low	147	3.66	1.43–9.37	0.007	0.09
	High	121	4.39	1.55–12.40	0.005	0.08
INR^a^	Low	142	3.28	1.25–8.58	0.02	0.15
	High	127	4.63	1.70–12.65	0.003	0.05
Partial thromboplastin time^a^	Low	132	2.79	1.05–7.42	0.04	0.28
	High	134	6.02	2.17–16.70	< 0.001	0.01
Platelet count^a^	Low	139	2.99	1.13–7.92	0.03	0.24
	High	130	5.80	2.06–16.35	< 0.001	0.02

a, These continuous variables were categorized as low (<median value) and high (≥median value). Median serum creatinine was 1.8 mg/dL and eGFR was 38 ml/min

*OR* Adjusted odds ratio, *CI* Confidence interval, *P* Significance values for adjusted odd ratio, *q* false discovery rate correction using the Benjamini-Hochberg method

## Discussion

In this retrospective study of patients undergoing percutaneous kidney biopsy, administration of desmopressin did not decrease bleeding events in patients with serum creatinine < 1.8 mg/dL, but was associated with increased odds of bleeding which was driven mainly by post biopsy drop in hemoglobin. Administration of desmopressin to patients with creatinine ≥1.8 mg/dL decreased the odds of bleeding.

Bleeding is the most common complication of percutaneous kidney biopsy [1, 9]. As compared to patients with normal kidney function, bleeding after kidney biopsy is encountered more frequently in patients with decreased kidney function [2, 5]. This is thought to be due to platelet dysfunction from multiple factors such as abnormal binding to the von Willebrand factor, platelet membrane abnormalities, uremic toxins such as guanidinosuccinic acid and phenolic acid which inhibit platelet aggregation and increase in prostacyclin and nitric oxide which also inhibits platelet aggregation [10, 11]. Desmopressin improves platelet aggregation by increasing release of von Willebrand factor and has been used to decrease bleeding after kidney biopsy. However, there is sparse data to support routine use of desmopressin and it is not known if there is a subset of patients who will benefit the most from administration of desmopressin. In our study administration of desmopressin to patients with elevated creatinine was associated with decreased bleeding risk. Similar findings were reported in study by Peters et al. in which administration of desmopressin to patients with serum creatinine > 150 μmol/L decreased post biopsy complications (3.4% vs 8.4%, OR 0.39, CI 0.17–0.90) [12]. On the other hand, in the study by Lim et al., there was no difference in bleeding when desmopressin was administered to patients with serum creatinine ≥150 μmol/L (15% vs 13.3%, *p* = 0.60) but patients who received desmopressin had more episodes of severe hyponatremia (10.4% vs 3%, *p* = 0.002) [13]. Based on findings of our study and the study by Peters et al. and Lim et al., a randomized controlled trial evaluating the efficacy of desmopressin for bleeding risk in patients with elevated creatinine is recommended.

Conversely, administration of desmopressin to patients with relatively preserved kidney function (serum creatinine < 1.8 mg/dL) did not show any benefit; on the contrary desmopressin given to patients with preserved kidney function was associated with increased odds of bleeding. Our findings are in contrast to study by Manno et al. in which administration of desmopressin resulted in decrease in post biopsy bleeding (13.7% vs 30.5%; relative risk, 0.45; 95% CI, 0.24–0.85; *P* < 0.01, 8). However, in the study by Manno et al., pre-biopsy administration of desmopressin did not show benefit in reducing hard clinical end points (such as erythrocyte transfusion, angiographic embolization to stop bleeding) and decrease in number and size of asymptomatic hematoma was the only benefit and clinical significance of this finding is not clear [14].

In the present study increased bleeding risk associated with desmopressin administration in low risk patients was driven mainly by decrease in post biopsy hemoglobin. Administration of desmopressin has been shown to decrease hematocrit [6]. It is possible that the decreased post biopsy hemoglobin in low risk patients may reflect this dilution effect and is not true bleeding from the kidney tissue. However, decrease in hemoglobin > 1 g/dL post kidney biopsy may lead to unnecessary further testing (extended period of observation, more blood draws to follow hemoglobin level, ultrasound or CT scan to look for hematoma) increasing health care costs and causing emotional trauma to patients. Desmopressin has also been associated with risks such as increased thrombotic risk as well as symptomatic hyponatremia [13, 15–18]. Of note, desmopressin was previously used in non-uremic patients undergoing major cardiac surgery to decrease bleeding [19]. However, it was associated with increased risk of thrombotic episodes and a meta-analysis showed no decrease in transfusion requirements and routine use of desmopressin for cardiac surgery is not recommended [20–23]. In the present study, desmopressin showed no benefit when administered to patients at low risk of bleeding (and has potential for harm) and routine administration of desmopressin in patients who are low risk is not recommended.

The present study includes a significant number of patients with elevated serum creatinine (> 70%), AKI (> 4%) and high BMI (> 40%) which is a strength of this study. Patient characteristics and biopsy technique reflect contemporary kidney biopsy practice which increases the generalizability of these results. This is a retrospective study and although we adjusted for known confounders, residual confounding is a limitation of the study. An 18 gauge biopsy needle was used in this study and the results may not be applicable to biopsy performed with 16 gauge needle. All patients in the study had normal bleeding time and coagulation parameters. Thus, the results of the study do not apply to patients with abnormal bleeding time or coagulopathy – in these patients, administration of desmopressin and correcting coagulation abnormalities prior to kidney biopsy is indicated. In our study, desmopressin was administered intravenously whereas in the study by Manno et al. [8] and Peters et al. [12], desmopressin was administered subcutaneously. When intravenous and subcutaneous administration of desmopressin was compared in patients with hemophilia, there was no difference in plasma desmopressin area under the curve, half-life or factor VIII coagulant activity [24, 25]. Based on these studies, it is unlikely that route of administration of desmopressin influences bleeding risk after kidney biopsy. Use of desmopressin was not associated with adverse events or symptomatic hyponatremia but the study was not powered for this indication.

## Conclusion

In summary, administration of desmopressin does not reduce clinically important bleeding in low risk patients undergoing kidney biopsy. Administration of desmopressin to high risk patients was associated with reduced odds of post-biopsy bleeding, a randomized controlled trial is needed to further evaluate the efficacy and safety of desmopressin in high risk patients.

## Data Availability

The datasets used and analyzed during the current study are available from the corresponding author on reasonable request.

## Abbreviations

AKI: Acute kidney injury
AUROC: Area under the curve
DDAVP: Desmopressin
eGFR: Estimated glomerular filtration rate
FDR: False discovery rate
gm/dL: Gram per deciliter
mg/dL: Milligram per deciliter
NOAC: Novel oral anticoagulant
OR: Odds ratio
PT: Prothrombin time
PTT: Partial thromboplastin time
RBC: Red blood cell
ROC: Receiver operating characteristic curve
SD: Standard deviation
μmol/L: Micromole per liter
